# Performance of Colorimetric Lateral Flow Immunoassays for Renal Function Evaluation with Human Serum Cystatin C

**DOI:** 10.3390/bios15070445

**Published:** 2025-07-11

**Authors:** Xushuo Zhang, Sam Fishlock, Peter Sharpe, James McLaughlin

**Affiliations:** 1 Nanotechnology Integrated Bioengineering Centre (NIBEC), Ulster University, Shore Road, Newtownabbey, County Antrim BT37 0QB, UK; s.fishlock@ulster.ac.uk; 2 Southern Health & Social Care Trust (SHSCT), Craigavon Area Hospital, 68 Lurgan Road, Portadown BT63 5QQ, UK; peter.sharpe@southerntrust.hscni.net

**Keywords:** gold nanoparticles, serum Cystatin C, lateral flow immunoassay, point-of-care

## Abstract

Chronic kidney disease (CKD) is associated with heart failure and neurological disorders. Therefore, point-of-care (POC) detection of CKD is essential, allowing disease monitoring from home and alleviating healthcare professionals’ workload. Lateral flow immunoassays (LFIAs) facilitate POC testing for a renal function biomarker, serum Cystatin C (CysC). LF devices were fabricated and optimised by varying the diluted sample volume, the nitrocellulose (NC) membrane, bed volume, AuNPs’ OD value and volume, and assay formats of partial or full LF systems. Notably, 310 samples were analysed to satisfy the minimum sample size for statistical calculations. This allowed for a comparison between the LFIAs’ results and the general Roche standard assay results from the Southern Health and Social Care Trust. Bland–Altman plots indicated the LFIAs measured 0.51 mg/L lower than the Roche assays. With the 95% confidence interval, the Roche method might be 0.24 mg/L below the LFIAs’ results or 1.27 mg/L above the LFIAs’ results. In summary, the developed non-fluorescent LFIAs could detect clinical CysC values in agreement with Roche assays. Even though the developed LFIA had an increased bias in low CysC concentration (below 2 mg/L) detection, the developed LFIA can still alert patients at the early stages of renal function impairment.

## 1. Introduction

The use of a home-based point-of-care (POC) approach for indicative purposes could date back to 1984 when the first pregnancy test was developed with hemagglutination inhibition [1]. In the 1980s, the pregnancy test was based on immunochromatography or enzyme immunoassay to detect human chorionic gonadotropin (hCG) in the urine [2]. The general device development challenge is that at higher concentrations the analyte would produce a test line intensity identical to a lower concentration’s test line signal intensity. This has been described previously concerning the linear detection range in the sigmoidal curve [3] with the application of the gold-carboxyl nanoparticles (AuNPs). Colloidal AuNPs, as a type of coloured NPs, are widely used in lateral flow immunoassays [4,5] since they are easy to prepare and functionalise [6].

Recent LFIAs are widely applied to disease diagnostics in animals and humans [7], covering the detection of viruses [8], proteins, bacteria [7], etc. Approaches for recognising and capturing these antigens precisely encompass the utilisation of antibodies, aptamers [9], and nanobodies, which are antibodies fragments containing variable heavy chain domains but without the fragment crystallizable region [10]. With a further broad selection of detection labels, including AuNPs [11], Au nanorods [12], Au nanoshells [13], and quantum dots [14], etc., the recent development of LFIAs involves the decoration of labels with nanozyme and oxidation for shorter result turnaround times [15]. These traits confer on the LFIAs a broad application in the POC field.

The prevalence of chronic kidney disease (CKD) in a UK population-based study is 18.2% among patients above 60 years old [16]. With a prevalence of 11% among Northern Ireland adults having CKD in a 2015 study, it is essential to evaluate the ability to monitor CKD at home or the hospital bedside for early diagnostic purposes [17]. Extensive evidence suggests that the prevalence of CKD affects 10% to 13% of the world population [18]. Further research in 2019 indicated the prevalence of CKD as 13.4% (between 11.7% and 15.1%) [19]. The POC assay has been established for monitoring disease at home and is strongly recommended by many governments [20,21]. This is especially important as CKD is related to important illnesses such as heart failure [22] and neurological disorders [23].

Cystatin C (CysC) is released by nucleated cells and has been utilised as a potential biomarker to rule out CKD [24]. Furthermore, CysC is related to inflammation pathways [25], and higher CysC indicates a stronger cardiac inflammatory response [26]. Moreover, CysC is indirectly illustrative of mild cardiac dysfunction, as mild cardiac dysfunction is associated with mild kidney dysfunction [27]. Thus, cardiac dysfunctions have a close correlation to renal malfunctions and vice versa. This leads to insufficient myocardial perfusion and severe clinical outcomes [28]. A higher CysC level is indicative of a high in-hospital readmission rate, poor cardiac recovery rate, and congestive heart failure prognostics [29]. Usually, the estimated glomerular filtration rate (eGFR) is an indication of renal function, and a lower eGFR has a higher possibility of all-cause mortality [30]. This work outlines the research regarding the development of such a POC technology and validates it via bench-top studies and some early-stage clinical evaluations.

## 2. Materials and Methods

### 2.1. AuNPs’ Characterisation and LFIA Fabrication

AuNPs’ characterisation was obtained via a field-emission scanning electron microscope (FESEM, SU5000, Hitachi High-Tech Europe GmbH, Krefeld, Germany). Imaging of AuNPs’ samples utilized the 10 kV acceleration voltage, with a working distance of approximately 6 mm, and magnifications of 110,000× and 401,000×, respectively. The main purpose of using the SEM was to check particle size, shape, and size spread, if any. Moreover, the characterisation of mAbs’ conjugation with AuNPs was described in the previous work [12] via dynamic light scattering and ultraviolet–visible spectroscopy, indicating the 89.5 µg/mL detector antibody is the optimum conjugation concentration for AuNPs.

Notably, the optical density (OD) is the unit of absorbance per unit length; it can be calculated as in Equation (1). L is the sample thickness, which is the distance the light travels through the sample; the I_0_ is the intensity of the incident beam of light, which is the light intensity before it passes through the sample. *I* is the light beam intensity after passing through the sample. *A_λ_* is the absorbance at the wavelength *λ* [31].

(1) $${OD}_{\lambda }=\frac{A_{\lambda }}{L}=\frac{1}{L}\;\log_{10}\left(\frac{I_{0}}{I}\right)$$

In total, 40 OD AuNPs (40 nm, ab269942, Abcam, Cambridge, UK) were conjugated with the 89.5 µg/mL detector mAbs (Fitzgerald, 10-7887, Stratech, Ely, UK). The conjugation instructions were thoroughly described [12] following the conjugation kit protocol in our previous study. Similarly, the conjugate pad and sample pad pre-treatment, together with AuNPs’ printing, were detailed in the previous work [12]. Moreover, the capture antibody (Fitzgerald, 10-7886) in 0.5 mg/mL and control line antibody goat anti-mouse IgG1 (Fitzgerald, 20R-IG003-FIT) in 1 mg/mL were dispensed at a printing speed of 1 µL/cm via Biodot (ZX1010, Chichester, UK) simultaneously on the CN95 NC membrane (UniSart, Sartorius, Goettingen, Germany) with a 0.5 cm gap in between.

**Figure 1:**
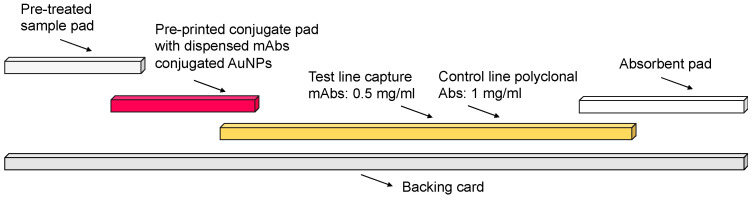
Lateral flow immunoassays’ (LFIAs) assembly that is used for human serum CysC quantification.

The LFIAs were assembled in order as shown in Figure 1, with 4 mm overlapping between the conjugate pad and NC membrane and 4 mm overlapping between the sample pad and conjugate pad. Notably, LFIAs are fully developed assays with four components, while the lateral flow dipstick (LFD) is a partially developed assay with only the NC membrane and absorbent pad [12].

### 2.2. Clinical Study Phases and Sample Selection Criteria

The ethics paperwork was conducted via the IRAS (Integrated Research Application System, IRAS Project ID 280849) website and approved with a favourable decision by the REC (Research Ethics Committee) ahead of the research study starting.

This study used fully anonymised human samples with no identifying demographic information or BMI (Body Mass Index); only samples’ accession numbers were recorded with their corresponding eGFR values and the stages of kidney function. The source of the samples was sufficient in numbers each day, coming from all Southern Health and Social Care Trust (SHSCT) hospital departments. Furthermore, the cycle of ‘freezing samples to thaw’ was monitored. All samples were discarded within 3 cycles of freeze-thaw [32]. Multiple freeze-thaw cycles would deteriorate the serum protein, thus leading to unreliable results.

The patients’ blood samples were taken from Craigavon Area Hospital over 6 weeks. Some of the patients were heart failure patients with mild or no kidney malfunction. Staff in Craigavon Area Hospital’s Clinical Molecular Laboratory performed the patients’ blood sample collections and centrifugation to acquire the serum. Each sample was taken from anonymised patient data at different stages of their kidney function, from normal to end-stage. Blood samples were spun down during routine clinical care via the laboratory’s centrifugation at 1300× *g* for 10 min [33]. Specifically, the inclusion and exclusion criteria were as follows:

Inclusion: Kidney disorder patients, 18 years and above, with known CysC values widely ranging from normal to end-stage kidney function.

Exclusion: Patients having high CysC values not related to kidney disorders. Patients under the age of 18 years, and samples that were suspected or confirmed with COVID-19.

In total, 558 fully anonymised samples were selected based on the calculated eGFR from creatinine. Individuals with ≥60 mL/min/1.73 m^2^ eGFR were classified as healthy and having normal kidney function in this study, unless there was the presence of other kidney damage, encompassing persistent proteinuria, haematuria, or structural kidney disease [16,17]. These 558 samples were initially assessed via the standard Roche CysC method to measure the exact levels of CysC concentrations. Then, the CysC samples (up to 310 from these 558 samples) with known CysC concentrations ranging from 0.49 mg/L to 7.16 mg/L were selected for LFIAs’ evaluation. In total, 310 patients’ samples (279 having no disease, 31 having disease) were run with LFIAs. Duplicate measurements were carried out for these 310 samples for the LFIAs’ study validation through processing the results with the mean and standard deviation.

A high prevalence of disease would require a smaller sample size. Conversely, to estimate the minimum sample size required, assuming the prevalence [34] of CKD was lower than the actual prevalence value of 10% [16]. The sample size could be calculated as a minimum sample size of 310 (hypothesis null to hypothesis alternative with a change from 0.7 to 0.9, reaching the power of 0.807 and *p*-value of 0.048) [34], as per the calculation being conducted via the statistical software PASS (Power Analysis and Sample Size 11. NCSS, Kaysville, UT, USA) and published in the Journal of Clinical and Diagnostic Research in 2016 [34]. Specifically, to reach the sensitivity and specificity of this diagnostic study, the pre-determined value needed to be at least 70% within the null hypothesis, indicating that the chance or probability of diagnosing a true positive or a true negative value was at least 70%. Additionally, power was the probability of rejecting the null hypothesis if the null hypothesis was false to make a correct decision. It also meant the probability of a test of significance could detect a deviation from the null hypothesis if the deviation existed. Generally, 0.8 or greater is necessary for finding a statistical significance if there is one. Thus, a sample size of 310 was chosen, and there was no optimum bias [35] during the sample collection stage.

This sample size also covered the specificity test’s minimum sample size requirement of 119, with 12 having the disease [34] (hypothesis null to hypothesis alternative with a change from 0.8 to 0.9, and the power of 0.819 with *p* as 0.04). The cited literature [34] stated numerous calculations for minimum sample sizes; therefore, a 310-sample size was chosen based on that literature, which is similar to the minimum sample size calculation as per the Daniel [32,36] formula, see Equation (2). Equation (2) suggests 240 samples as a minimum sample size to align with 10% prevalence and 5% precision with 99% confidence. Equation (2)’s application for the minimum sample size calculation was also used in the pandemic study [32]:

(2) $$n=\frac{Z^{2}P\;(1-P)}{d^{2}},\;$$

where n is the minimum sample size, Z is the statistic chosen based on a confidence level (99% confidence, Z = 2.58), *P* is the estimated prevalence, and d is precision (5%).

To validate the in-house developed LF system against a standard, in terms of CysC quantitative measurements and detections, 3 phases of clinical studies were conducted.

#### 2.2.1. Phase 1 Study

Phase 1 preliminary trials involved 40 serum samples. Patients’ samples were processed by biomedical scientists in the Clinical Biochemistry Laboratory in Craigavon Area Hospital to extract serum from the whole blood and analyse a Urea and Electrolytes (U&E) profile, which included the eGFR measurement based on creatinine. Notably, the serum of each sample obtained after centrifugation was immediately frozen and stored at −20 °C or −80 °C (for long-term storage) [37]. At Craigavon Southern Trust, the samples were picked up from a 4 °C cold room, then frozen and stored at −80 °C after CysC measurements. LFIAs’ strips were printed and assembled just a day before the LFIAs to ensure there were no ageing or environmental storage effects on the strips.

**Figure 2:**
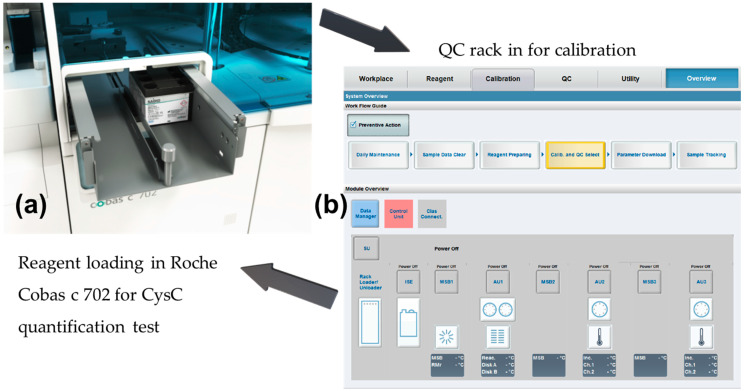
(**a**) Analyser Cobas c 702 reagents loading for CysC quantification; (**b**) Quality control (QC) and system calibration workstation of Roche CysC test. By comparing the results of Southern Trust and Belfast Trust, whether the Roche standard has reached its QC validation and whether the methods are reliable can be verified.

The following procedure was followed:

1. As there was no difference in diagnostic values for serum CysC and plasma CysC, the level of serum CysC was also used to indicate the level of plasma CysC unless otherwise stated [38]. The 40 serum CysC samples were quantified with the standard via the Roche Cobas 8000 Modular Series (Cobas c 702 modules, Roche, Basel, Switzerland) to reveal CysC concentrations.
2. For these 40 samples, a further measurement was conducted in Belfast Health & Social Care Trust, and standard deviations between the results were recorded. Details of the procedures and diagrams are shown in Figure 2.

The Roche Cobas c8000 modular analyser series with analyser Cobas c 702’s test principle is a particle-enhanced immunoturbidimetric assay. The human CysC agglutinated with the latex particles in glycine buffer coated with anti-CysC antibodies (rabbit). Then, the aggregate was determined at 546 nm turbidimetrically [39]. The system is a fully automated system for CysC quantification. The intensity of the light read at 546 nm was proportional to the CysC concentration in the sample. The assay used a six-point calibrator C.f.a.s. Cystatin C, with a measuring range between 0.4 mg/L and 6.8 mg/L [40]. Subsequently, the stability of the CysC level was tested to observe the serum CysC level over a week to observe any changes when stored at 4 °C within a cold room.

All samples were measured for CysC values before and after running the quality control from the analyser. Specifically, the precision was determined by 3 Roche diagnostic quality control regents with CysC concentrations of 1.10 mg/L CysC, 1.62 mg/L, and 4.48 mg/L, respectively. These 3 control reagents were run before and after the quantification of the CysC level to ensure the validity of the measurement results.

#### 2.2.2. Phase 2 Study

Phase 2 adjustments of LFIAs were conducted based on the Roche method’s CysC concentration results. A Lumos reading of recombinant CysC protein (extracted from human, Fitzgerald, 30R-3194, Stratech, Ely, UK) was used for LFIAs’ optimisations. The spiked protein CysC (30R-AC035, Fitzgerald, Stratech, Ely, UK) at 0.050 mg was spiked into 5 mL CysC-depleted serum (HyTest Biotechnology, Turku, Finland) as a 10 mg/L stock solution. The solution was diluted into 8 mg/L, 5 mg/L, 2.5 mg/L, 1 mg/L, 0.5 mg/L, and 0 mg/L for obtaining the calibration curve. The serum samples were further diluted 36-fold in the PBS medium for evaluation [12].

Optimisation involved the selection of the NC membrane. Furthermore, the diluted sample volume and bed volume optimisation were conducted, and the OD values of conjugated AuNPs, as well as their volume, were adjusted accordingly. In the interim, the suitable dilution factor for LFD and LFIAs was demonstrated. The optimised LFIAs’ system was then applied with human serum for the phase 3 study’s evaluation.

#### 2.2.3. Phase 3 Study

**Figure 3:**
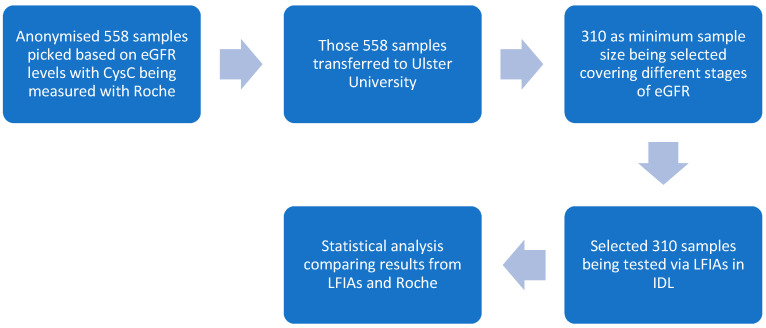
The detailed protocol for this study is provided. IDL is short for Integrated Diagnostics Lab. The samples with known eGFR were processed by Roche with corresponding quality control to obtain the CysC concentrations. Selected samples were used to evaluate the built LFIAs.

Phase 3 trials with optimised LFIAs as shown in Figure 3:

1. In total, 558 serum samples were tested via the Roche standard. (Note: Some of the previous 40 samples were selected and tested with the optimised LFIAs to reach the total sample amount of 310).
2. With the 558 samples, patients were defined as having serum CysC values above 1.02 mg/L [41], as this was the level of CysC in healthy volunteers [42,43].
3. In total, 310 samples were tested on LFIAs, and test and control line signal intensities were documented by the Lumos reader (Lumos Diagnostics, San Diego, CA, USA), with the optimised settings.

The sensitivity of the study could be addressed by 31 samples having the disease out of 310. The specificity of the assays and whether the LF results were affected by interfering biomarkers could also be elucidated as long as there were 26 samples having the disease out of 257. The minimum sample size calculation was conducted via the PASS software [34]. With more than a thousand proteins in the peripheral blood [44], interfering proteins like C-reactive protein [45], and rheumatoid factor [46] might or might not interfere with LFIAs’ performances.

## 3. Results

### 3.1. AuNPs’ Characterisation

**Figure 4:**
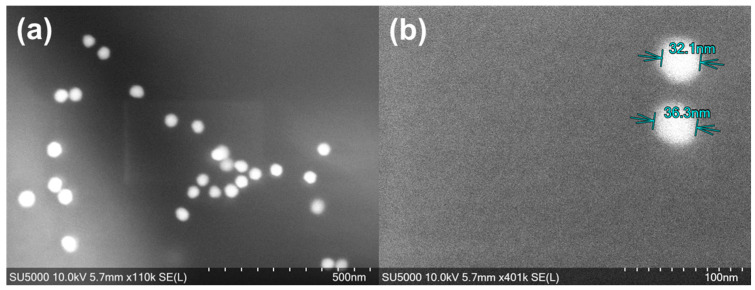
SEM of (**a**) AuNPs in 10 kV voltage, ×110 k magnification; (**b**) AuNPs’ size distribution in 10 kV voltage in ×401 k magnification.

For 40 nm AuNPs, the spherical solid sphere of AuNPs is shown in Figure 4a by having a bright circular shape in the SEM image. The AuNPs look round-shaped, and the diameter is distributed uniformly (Figure 4b), which encourages colour consistency due to their unique plasmonic traits. The characterisation results for the ideal detector antibody concentrations for AuNPs’ conjugation were presented in previous work [12].

### 3.2. Optimal OD Value and NC Membrane for AuNPs’ LFIAs

**Figure 5:**
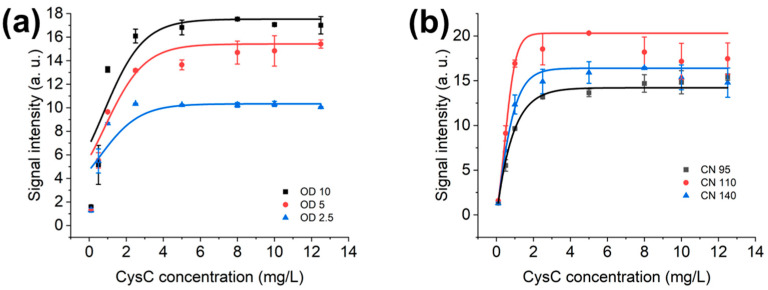
The 1 μL sample volume with 5 μL AuNPs’ conjugated mAbs and 54 μL running buffer calibration curves in (**a**) OD values’ optimisation in regard to OD 10, OD 5, OD 2.5; (**b**) calibration curves with CN 95, CN 110, and CN140 (OD 5 AuNPs).

AuNPs and their respective optimised OD values are detailed in Figure 5a. Accordingly, the linear range of OD 10 is up to 2.43 mg/L CysC; OD 5 has a linear range of up to 2.51 mg/L CysC; OD 2.5 has a linear range of up to 2.21 mg/L CysC. OD 5 provides the widest linear range among OD 10 and 2.5. Thus, OD 5 is selected.

With faster liquid wicking and wetting, CN95 could detect CysC with less sensitivity than CN110 but with a broader linear range compared to CN110. The linear range of CN 95 is up to 2.51 mg/L, with CN 110 up to 0.91 mg/L, and CN 140 up to 0.63 mg/L. Thus, CN95 is selected due to its wider linear detection range.

### 3.3. Optimisation of LFIAs’ Bed Volume and AuNPs’ Volume in LFD

**Figure 6:**
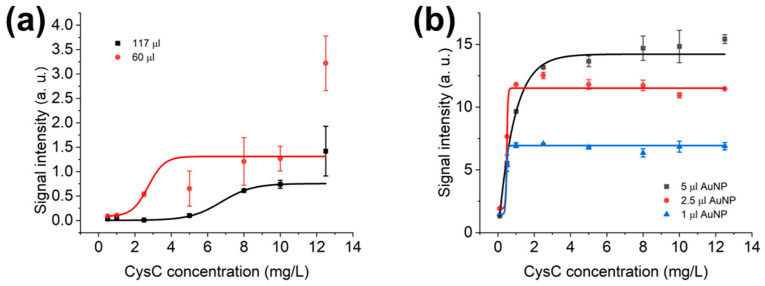
(**a**) AuNPs in LFIAs’ bed volume comparison, sample volume: 2.5 μL; (**b**) AuNPs OD 5 volume optimisation calibration curves with 5 μL, 2.5 μL, and 1 μL AuNPs, sample volume: 1 μL.

In Figure 6a, the whole LFIAs are assembled, and a running buffer volume is optimised with a 60 μL running buffer volume being selected. Even 60 µL bed volume has a linear detection range from 2.05 mg/L to 3.41 mg/L, while 117 µL bed volume detects from 5.42 mg/L to 8.08 mg/L. This suggests that a larger volume of running buffer could generate a linear detection range suitable for higher concentrations of analyte, but at the same time extend the results’ turnaround time. More specifically, it takes 10 min for a 60 μL bed volume LFIA to be ready for results’ interpretation, while a 117 μL bed volume requires a minimum of 12 min for even buffer distribution. To have a trade-off between the result’s turnaround time and detection linear range, a 60 µL bed volume is chosen.

Meanwhile, the gold nanomaterials’ volumes of 5 µL, 2.5 µL, and 1 µL are optimised as seen in Figure 6b. The 5 μL volume of mAbs-conjugated gold nanomaterials would provide the ideal outcome by comparing 2.5 μL and 1 μL volumes. Specifically, 5 μL AuNPs OD 5 could detect up to 2.51 mg/L, while a volume of 2.5 μL could detect from 0.44 mg/L to 0.54 mg/L, while 1 μL could detect from 0.41 mg/L to 0.52 mg/L as the linear range. This makes the 5 μL volume ideal for next-stage optimisation, as 5 µL OD 5 can linearly detect the highest CysC concentration (2.51 mg/L) among 2.5 µL (0.54 mg/L) and 1 µL (0.52 mg/L). The sigmoidal curve fitting in Figure 6b is conducted for the CysC from 0.1 mg/L to 12.5 mg/L, and the linear range is from 0.1 mg/L to 2.5 mg/L CysC, as test line signal intensities are levelled up from 2.5 mg/L CysC onwards.

### 3.4. Optimisation of Sample Dilution Factor and Diluted Sample Volume in LFD and LFIAs

**Figure 7:**
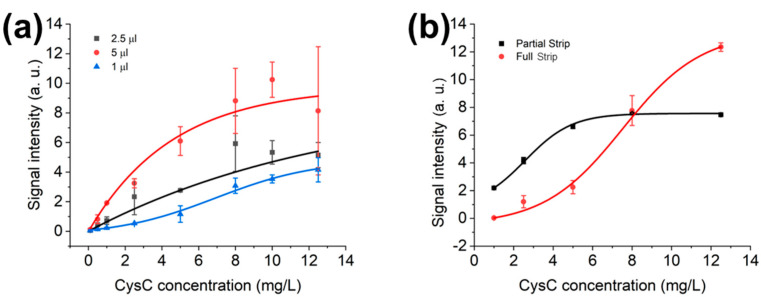
(**a**) Samples in 100-fold sample dilution factor, the sample volume optimisation calibration curves; (**b**) LFIAs and LFD with running buffer volume of 60 μL, sample in 1.5-fold dilution with sample volume of 2.5 μL.

Optimisations use 1 μL sample volume. However, 1 μL is a small volume for pipetting and adding to the system; therefore, further optimisation using 100-fold dilution is carried out, but with 5 μL, 2.5 μL, and 1 μL sample volumes. In Figure 7a, a 100-fold dilution is utilised, and sample volumes vary from 1 μL, 2.5 μL, and 5 μL. As the nationwide human serological study with LFIAs [32] utilised the 2.5 μL sample volume instead of 1 μL, 2.5 µL is selected, even with the linear range of 1 µL sample volume, at 100-fold dilution, detecting from 2.73 mg/L to 11.12 mg/L. Furthermore, 2.5 μL is picked because the pipette precision relies on the air displacement, which is between the position and the liquid being dispensed. The larger the air displacement is, the closer the volume and the desired amount will be.

The assembly of the conjugate pad and sample pad does interfere with the optimised setup of the existing LFD, as shown in Figure 7b. The performance of CysC detection between the dipstick format LFD and full LFIAs is different. The experiments have been carried out with 1.5-fold sample dilution and 60 μL running buffer in partial LFD and full LFIA strips in Figure 7b. Partial strips have a linear detection range of up to 4.41 mg/L, with the full strip having a linear detection range from 4.46 mg/L to 10.61 mg/L.

### 3.5. Evaluation of the LFIAs’ Performance with Roche Standard

Table 1 lists the concentrations of spiked CysC in the depleted serum in the left column, with the average test line intensities in the second column measured via LFIAs, and the corresponding standard deviation in the third column.

Based on the calibration curve generated from Table 1, the LFIAs’ test line intensities’ results are read from the calibration curve to obtain the required CysC concentration values. In other words, Table S1 LFIAs’ results are obtained by interpolating the test line signal intensity readings with the calibration curve; the corresponding CysC concentrations are then calculated. This is realised by applying the fully anonymised 2.5 µL human samples in a 36-fold dilution factor on the sample pad. These CysC concentration values are then compared with Roche’s results from the SHSCT. See Table S1 in the Supporting Information.

**Figure 8:**
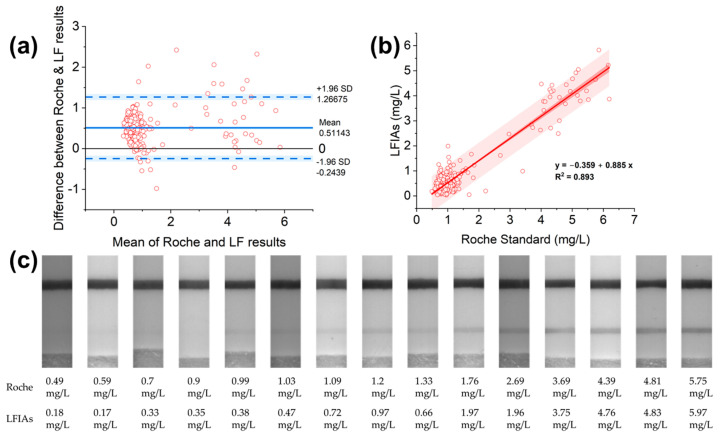
(**a**) Bland–Altman plot of comparison of the methods of Roche and LFIAs; (**b**) linear correlation results obtained by both LFIAs and the reference method Roche for CysC concentration quantification. The linear regression analysis yields a correlation between methods ($y=-0.359+0.885x,\;R^{2}=0.893,\;n=310$); (**c**) LFIAs images with the reference Roche CysC concentrations.

The quantitative measurements of CysC via LFIAs with the read-out concentration given by the high-sensitivity camera based on the OD of the test line are compared with measurements from the Roche standard. The comparison is indicated via the Bland–Altman analysis in Figure 8a. The Bland–Altman plot is generated with the Y axis as the CysC concentration difference between the Roche and LFIAs results, with the X axis as the mean CysC concentration values of the two methods; the original data can be referred to in Table S1. In total, 95% of the data should be within the mean ± 1.96 standard deviations (limits of agreement) [47] (blue lines in Figure 8a). With 16 data points of 310 data points outside the mean ± 1.96 standard deviation, 294 data points, which amount to 95% of the data points, lie within the limits of agreement. The plot has densely plotted points at below 2 mg/L CysC mean values but sparsely plotted points at above 2 mg/L mean concentration CysC values. Figure 8b shows the regression analysis of the LFIAs and the standard Roche method. The slope of the regression equation (0.885) and Pearson’s r (0.945) are all close to 1, suggesting a good correlation between the methods of LFIAs and the reference Roche. Figure 8c depicts the Roche CysC ranging from 0.7 mg/L to 4.39 mg/L measured by LFIAs.

Additionally, 558 samples are selected based on their eGFR values, as in Table 2, third column. The criteria of the eGFR categories and the corresponding sample amount selected for the LFIAs’ tests are summarised in Table 2’s columns.

## 4. Discussion

### 4.1. Optimisation of the LF System

In Figure 5a, OD is determined at the peak wavelength of gold nanomaterials’ surface plasmon resonance (40 nm AuNPs: 530 nm [49,50]). The concentration is calculated with this value using Beer’s Law [51]. Similarly, with increasing OD values, the test line signal intensities are strengthened. As there are higher volumes and amounts of mAbs-bound AuNPs in the system, the test line intensity will increase as long as the immobilised capture antibody on the membrane has not been saturated by the mAbs-bound AuNPs. Furthermore, AuNPs can be less sensitive regarding the limit of detection, and this is improved by another colorimetric nanomaterial, cerium oxide NPs [15]. This type of nanozyme can oxidise the organic substrate, generating colorimetric signals within 3 min in the detection of the ng/mL range of C-reactive protein.

In Figure 5b, NC membrane optimisation, fast flow membrane CN95 could extend the detection range to higher CysC concentration levels compared with CN110. CN110 has narrower pores compared with CN95. This leads to a longer wicking time and higher sensitivity. The smaller the pore size, the greater the back pressure that is generated, which prevents the flow towards the absorbent pad by holding on to the liquid [52]. Since the development of CysC is a quantitative assay instead of a qualitative assay, the sensitivity is considered with a trade-off against the detection range.

Moreover, the reading time is crucial in LF results’ interpretation. Longer waiting times will allow the full saturation of the membrane, thus leading to a set of stronger test line intensities (see Figure 5b). This is in accordance with research applying the fluorescence approach for CysC detection [53]. However, a longer waiting time would deteriorate the purpose of the point-of-care, plus, the test line intensities drop dramatically when analyte concentrations are above 5 mg/L with the application of CN110 (Figure 5b). Moreover, the dual-model LFIAs utilising the AuNPs and quantum dots for CysC detection can provide results in 8 min. However, the detection sensitivity is 0.61 ng/mL, which may alarm individuals who are healthy without CKD [14].

As in Figure 6a, the bed volume is the total volume of the liquid required to wet out the material [54]. The bed volume is closely related to the thickness of the material, and researchers investigate the bed volume for each LFIA component and presume that larger bed volumes for a sample pad would allow more AuNPs with slower flow and possibly higher sensitivity [55]. A similar trend is observed, with higher density of the AuNPs, and the test line signal intensity is enhanced [56], as in Figure 6b.

Figure 7a indicates that an extra sample amount enhances the test line signal intensities. Similar results are obtained from TSH (Thyroid-Stimulating Hormone) detection with increasing sample volumes from 10 µL to 50 µL [13].

### 4.2. Developed LFIAs’ Performance in Comparison with the Roche System

The comparison of the methods of Roche and LFIAs is conducted via the Bland–Altman analysis. The concentrations as determined via the developed LFIAs demonstrate a high correlation with the Roche standard, particularly at higher concentrations, as the plotted points are sparser at the higher concentration range in the Bland–Altman plot. Correlation differences begin to increase when the concentrations are lower, as the plotted points are denser at the lower concentration range in the Bland–Altman plot. This can be seen from the Bland–Altman plot, as plotted points are less scattered at lower concentrations but scattered at higher concentrations [47,57].

Moreover, the mean value of 0.51 in Figure 8a suggests that the LFIAs measure 0.51 units less than the Roche standard. The 95% confidence intervals of the mean and limits of agreement (dashed lines) are calculated in Table 3. As seen in Table 3, the Roche method may be 0.24 units below the LFIAs’ results or 1.27 units above the LFIAs [57]. However, the Bland–Altman plot does not show if the agreement is sufficient or suitable to use one method or the other; generally, it simply quantifies the bias and limits of agreement. It is possible to say the bias is significant as the line of equality is not within the 95% confidence intervals of the mean difference (from 0.47 to 0.55). However, only biological, analytical, and clinical goals would be able to define whether the agreement interval is too wide or sufficiently narrow for device development purposes [57].

Figure 8c shows differences in the Roche and LFIAs’ measurement results. As the concentration of the capture antibody being printed as a test line is 0.5 mg/mL, increasing the capture antibody on the test line from 0.5 mg/mL but not exceeding 1 mg/mL could improve the detection of lower concentration samples. Potentially, increasing the printed test line antibody’s concentration could tackle the underestimation of the CysC values by LFIAs [12]. Similar results were obtained in cardiac troponin 1 detection with superparamagnetic nanobeads, by increasing the test line antibody amount from 1 to 3 mg/mL [58]. Moreover, to mitigate the differences between the Roche and LFIAs, larger diameter AuNPs can produce a stronger test line signal intensity compared with smaller diameter AuNPs at the identical density [56], making them capable of generating less-faint test lines, thus corresponding to higher CysC values. In addition, fluorescent gold nanorods enhance the detection limit compared with AuNPs [59], encouraging a stronger test line at a minimal level of CysC. The developed LFIAs in this study have a detection limit [12] of 0.98 mg/L from the sigmoidal curve in Equation (3), while other work [11] has enhanced the detection limit of 0.18 mg/L CysC. However, the 0.18 mg/L detection limit could result in false positives, thus alerting healthy individuals without CKD. Furthermore, this work uses 0.5 mg/mL less immobilised capture antibody than our previous work [12], which confers a reduced production cost and is thus friendly for the market. Similarly, other work [53] with fluorescent LFIAs for CysC detection would incur the cost of the reader, as an excitation light source is essential to interpret the fluorescent test line signals. While some commercial products [60] fail to provide assay specificity and sensitivity, this work highlights the CysC LFIAs’ development and thus lays the foundation for future product refinement.

(3) $$y=\frac{4.149}{1+e^{\left(-0.556\right)\times \left(x-4.618\right)}}$$

Within the 558 human serum samples, CysC ranges from 0.49 mg/L to 7.16 mg/L. The LFIAs are optimised to allow the CysC detection to be undetectable at 0.49 mg/L CysC level [34,61] with the application of AuNPs. The lowest CysC concentration (among 558 patients) of 0.49 mg/L is present by the Roche analysis as the lowest CysC value among the 558 samples. LFIAs could not detect this value satisfying the assay development.

Typically, urine CysC has a lower concentration in contrast with serum CysC, although it is better at predicting renal tubular dysfunction [62]. However, the serum CysC level is closely related to acute kidney injury and cardiovascular risks [63]. Thus, the combination of urinary CysC and serum CysC monitoring should be beneficial to POC disease monitoring.

### 4.3. Developed LFIAs’ Sensitivity and Specificity Evaluation

The healthy range of serum CysC has been reported as 0.62–1.02 mg/L from a study with 200 healthy blood donors, with a specification that male CysC (0.71–1.02 mg/L) is higher than female CysC (0.58–0.98 mg/L) [64]. The cut-off level of serum CysC less than or equal to 1.02 mg/L is referenced as healthy in this study. The calculated [65] sensitivity of the developed LFIAs is 96.8% (95% CI 90.5%, 100%), with a specificity of 94.6% (95% CI 91.9%, 97.3%).

## 5. Conclusions

This study designed and developed the LFIAs with AuNPs for human serum sample evaluation. This work also provided the potential for developing the POC device used for CKD monitoring at home, thus alerting patients at the initial stage of renal impairment. This, on the other hand, indirectly reduces the patients’ hospital visit frequency and mitigates the healthcare professionals’ workload. Since the developed LFIA device had a good correlation with the Roche standard at lower concentrations of CysC, it has achieved the goal of raising awareness among patients with mild renal deterioration at the initial and early stages. The developed LFIA measured 0.51 mg/L CysC, less than the standard method, the Roche assay. However, with 95% of the data points lying within the limit of agreement, this suggested there was an agreement between LFIAs and the Roche test, even if the bias was significant. The bias was lower when detecting the high concentration of CysC, while the bias was higher in low concentrations of CysC, especially below 1 mg/L. However, the developed LFIAs excluded the matrix effect interference and calibration error.

## Figures and Tables

**Table 1:** The calibration curve, with the concentration of each data point in the first column, average Lumos LFIA readings in the second column, and standard deviation in the third column.

Conc. mg/L	Average (Arb. Units)	Standard Deviation (SD)
10	4.1492	0.511
8	3.5951	0.638
5	1.9450	0.807
2.5	1.5243	0.326
1	0.6039	0.171
0.5	0.3416	0.324
0.1	0.3370	0.225
0	0.1861	0.099

**Table 2:** The categories or stages of CKD and corresponding eGFR value classifications [48]. Correspondent sample amounts tested via Roche and LFIAs are listed.

Stages of CKD			
Kidney Function	GFR (mL/min/1.73 m^2^)	Samples’ Distribution Measured by Roche	Samples Distribution Measured by LFIAs
1	≥90 *	279	279
2	60–89 *		
3a	45–59	112	0
3b	30–44		
4	15–29	126	10
5	<15/under dialysis	41	21

* Not CKD unless haematuria, abnormal pathology, and structure exist.

**Table 3:** Bland–Altman statistics from Table S1 data, including the confidence intervals’ calculation.

Parameter	Unit	Standard Error Formula	Standard Error (Se)	*t* Values for 309 Degrees of Freedom	Confidence (Se × t)	Confidence Intervals from	Confidence Intervals to
sample size (n)	310						
degrees of freedom (n − 1)	309						
difference mean (ū)	0.51	$\sqrt{s^{2}/n}$	0.02	1.97	0.04	0.47	0.55
standard deviation (SD)	0.39						
ū − 1.96 s	−0.24	$\sqrt{3s^{2}/n}$	0.04	1.97	0.08	−0.32	−0.16
ū + 1.96 s	1.27	$\sqrt{3s^{2}/n}$	0.04	1.97	0.08	1.19	1.35

## Data Availability

Data are contained within the article and Supplementary Materials.

## Supplementary Materials

The following supporting information can be downloaded at: https://www.mdpi.com/article/10.3390/bios15070445/s1, Table S1: 310 human serum samples for Bland–Altman analysis.

## Abbreviations

The following abbreviations are used in this manuscript:

CKD	Chronic kidney disease
POC	Point-of-care
LFIA	Lateral flow immunoassay
LF	Lateral flow
NC	Nitrocellulose
NP	Nanoparticle
OD	Optical density
hCG	Human chorionic gonadotropin
eGFR	Estimated glomerular filtration rate
PBS	Phosphate buffered saline
BSA	Bovine serum albumin
LFD	Lateral flow dipstick
IRAS	Integrated Research Application System
REC	Research Ethics Committee
BMI	Body mass index
QC	Quality control
IDL	Integrated diagnostics lab
SD	Standard deviation
TSH	Thyroid-stimulating hormone
FESEM	Field-emission scanning electron microscope
mAbs	Monoclonal antibodies
