# Supplementary material for: Performance of Colorimetric Lateral Flow Immunoassays for Renal Function Evaluation with Human Serum Cystatin C
Source: Biosensors (Basel). 2025 Jul 11;15(7):445. doi: 10.3390/bios15070445 (PMC12293487; doi:10.3390/bios15070445)
Supplement: Supplementary file 1 [file biosensors-15-00445-s001.zip › biosensors-3678629-supplementary.pdf]

Supporting Information

Performance of Colorimetric Lateral Flow Immunoassays for Renal Function Evaluation with Human Serum Cystatin C

Xushuo Zhang [1,*], Sam Fishlock [1], Peter Sharpe [2], James McLaughlin [1,*]

[1] Nanotechnology Integrated Bioengineering Centre (NIBEC), Ulster University, Shore Road, Newtownabbey, County Antrim, BT37 0QB, UK; s.fishlock@ulster.ac.uk (S.F.)

[2] Southern Health & Social Care Trust (SHSCT), Craigavon Area Hospital, 68 Lurgan Road, Portadown, BT63 5QQ, UK; peter.sharpe@southerntrust.hscni.net (P.S.)

***Corresponding Authors: x.zhang@ulster.ac.uk (X.Z.); jad.mclaughlin@ulster.ac.uk (J.M.)**

Table S1: 310 human serum samples for Bland-Altman analysis

Table S1 The accession number of the samples, plus their corresponding Roche method results and LFIAs results. Samples were pre-processed within the Clinical Biochemistry Laboratory, Craigavon Area Hospital, then all samples were stored in Craigavon Area Hospital before being transported to Ulster University where LFIAs were carried out later.

Sample accession number	Roche (mg/L)	LFIAs (mg/L)	Standard deviation (SD) of LFIAs	Mean of Roche and LFIAs	Difference between Roche and LFIAs
2205105001	1.09	0.37	0.498	0.73	0.72
2205105002	0.98	0.37	0.367	0.67	0.61
2205105004	0.73	0.35	0.184	0.54	0.38
2205105009	0.79	0.59	0.128	0.69	0.20
2205105014	0.94	0.11	0.144	0.52	0.83
2205105015	1.14	0.73	0.992	0.94	0.41
2205105016	0.73	0.77	0.186	0.75	-0.04
2205105017	1.23	0.30	0.250	0.77	0.93
2205105018	0.77	0.35	0.326	0.56	0.42
2205105020	1	0.40	0.011	0.70	0.60
2205105021	0.62	0.48	0.391	0.55	0.14
2205105022	1.04	0.18	0.235	0.61	0.86
2205105023	0.76	0.70	0.749	0.73	0.06
2205105028	0.9	0.23	0.168	0.57	0.67
2205105029	0.84	0.25	0.284	0.55	0.59
2205105032	0.7	0.30	0.042	0.50	0.40
2205105033	0.82	0.17	0.147	0.49	0.65
2205105038	0.86	0.29	0.016	0.57	0.57
2205105039	1.22	0.18	0.024	0.70	1.04
2205105040	0.98	0.15	0.030	0.56	0.83

2205132228	0.72	0.18	0.031	0.45	0.54
2205132229	1.01	0.37	0.175	0.69	0.64
2205132230	0.84	0.70	0.160	0.77	0.14
2205132231	0.9	0.56	0.064	0.73	0.34
2205132232	1.28	0.52	0.511	0.90	0.76
2205132233	0.59	0.27	0.132	0.43	0.32
2205132234	1.07	0.38	0.005	0.72	0.69
2205132235	1.04	0.52	0.577	0.78	0.52
2205132236	1.01	1.99	0.131	1.50	-0.98
2205132237	0.99	0.37	0.212	0.68	0.62
2205132238	1.35	0.87	0.348	1.11	0.48
2205132239	0.8	0.56	0.613	0.68	0.24
2205132240	1.43	0.89	0.339	1.16	0.54
2205132241	1.09	1.19	1.224	1.14	-0.10
2205132242	1.85	1.22	0.906	1.54	0.63
2205132243	0.9	0.38	0.413	0.64	0.52
2205132244	0.77	0.21	0.298	0.49	0.56
2205132245	0.79	0.07	0.057	0.43	0.72
2205132246	0.8	0.40	0.479	0.60	0.40
2205132247	0.86	0.47	0.310	0.66	0.39
2205132248	0.76	0.40	0.047	0.58	0.36
2205132249	0.84	0.14	0.159	0.49	0.70
2205132250	0.74	0.22	0.298	0.48	0.52
2205132251	0.77	0.77	0.482	0.77	0.00
2205132252	0.69	0.76	1.035	0.73	-0.07
2205132253	0.78	0.23	0.298	0.51	0.55
2205132254	1.17	0.76	0.124	0.97	0.41
2205132255	0.87	0.32	0.199	0.59	0.55
2205132256	0.87	0.54	0.416	0.71	0.33
2205132257	1.03	1.55	0.113	1.29	-0.52
2205132258	0.95	0.59	0.056	0.77	0.36
2205132259	0.84	0.18	0.150	0.51	0.66
2205132260	0.8	0.30	0.249	0.55	0.50
2205132261	0.65	0.08	0.069	0.37	0.57
2205132262	0.84	0.31	0.137	0.58	0.53
2205132263	0.87	0.33	0.290	0.60	0.54
2205132264	0.84	0.26	0.067	0.55	0.58
2205132265	0.74	0.11	0.070	0.43	0.63
2205132266	1.11	0.30	0.139	0.71	0.81
2205132267	0.93	0.81	0.385	0.87	0.12
2205132268	0.97	0.71	0.207	0.84	0.26
2205132269	0.88	0.53	0.054	0.71	0.35
2205132270	0.77	0.47	0.358	0.62	0.30
2205132271	0.96	0.39	0.200	0.68	0.57
2205132272	0.65	0.22	0.169	0.43	0.43

2205132273	0.87	0.58	0.090	0.73	0.29
2205132274	0.82	0.39	0.283	0.61	0.43
2205132275	0.73	0.46	0.084	0.60	0.27
2205132276	1.04	0.39	0.537	0.71	0.65
2205132277	0.83	0.40	0.013	0.61	0.43
2205132278	1.34	0.47	0.383	0.91	0.87
2205132279	1.06	0.65	0.407	0.85	0.41
2205132280	0.98	0.30	0.208	0.64	0.68
2205132281	2.69	1.62	0.490	2.15	1.07
2205132282	1.2	0.63	0.478	0.92	0.57
2205132283	1.13	0.37	0.071	0.75	0.76
2205132284	0.8	0.13	0.038	0.46	0.67
2205132285	1.06	0.81	0.079	0.93	0.25
2205132286	0.85	0.41	0.079	0.63	0.44
2205132287	1.05	0.61	0.116	0.83	0.44
2205132288	1.09	0.64	0.558	0.87	0.45
2205132289	1.05	0.65	0.074	0.85	0.40
2205132290	0.76	0.41	0.358	0.59	0.35
2205132291	2.97	2.47	0.210	2.72	0.50
2205132292	1.18	0.70	0.184	0.94	0.48
2205132293	0.63	0.23	0.053	0.43	0.40
2205132294	1.11	0.47	0.526	0.79	0.64
2205132295	0.89	0.44	0.004	0.67	0.45
2205132296	1.05	1.35	0.158	1.20	-0.30
2205132297	0.97	0.31	0.095	0.64	0.66
2205132298	0.96	0.16	0.029	0.56	0.80
2205132299	0.88	0.71	0.189	0.79	0.17
2205132300	1.04	0.44	0.160	0.74	0.60
2205132301	0.91	0.60	0.180	0.76	0.31
2205132302	0.79	0.36	0.010	0.58	0.43
2205132303	1.23	0.49	0.297	0.86	0.74
2205132304	0.82	0.20	0.047	0.51	0.62
2205132305	0.89	0.32	0.237	0.61	0.57
2205132306	0.77	0.36	0.125	0.56	0.41
2205132307	1.18	0.15	0.142	0.67	1.03
2205132308	0.8	0.45	0.018	0.62	0.35
2205132309	1.07	0.32	0.192	0.69	0.75
2205132310	0.77	0.67	0.020	0.72	0.10
2205132311	1.17	0.52	0.131	0.84	0.65
2205132312	1.08	0.18	0.006	0.63	0.90
2205132313	1.17	0.15	0.029	0.66	1.02
2205132314	1.29	0.69	0.746	0.99	0.60
2205132315	1.19	0.43	0.442	0.81	0.76
2205132316	0.9	0.09	0.064	0.50	0.81
2205132317	0.82	0.30	0.183	0.56	0.52

2205132318	0.95	0.54	0.252	0.74	0.41
2205132319	0.9	0.21	0.176	0.56	0.69
2205132320	0.91	0.67	0.317	0.79	0.24
2205132321	1.11	0.77	0.401	0.94	0.34
2205132322	0.72	0.48	0.056	0.60	0.24
2205132323	0.83	0.19	0.072	0.51	0.64
2205132324	1.82	0.72	0.184	1.27	1.10
2205132325	0.83	0.30	0.358	0.57	0.53
2205132326	0.76	0.39	0.329	0.57	0.37
2205132327	0.73	0.50	0.469	0.62	0.23
2205169789	0.89	0.35	0.181	0.62	0.54
2205169790	2.22	0.19	0.037	1.21	2.03
2205169798	0.93	0.44	0.065	0.69	0.49
2205169799	1.13	0.68	0.186	0.90	0.45
2205175109	0.84	0.08	0.028	0.46	0.76
2205175114	1.58	1.09	0.353	1.33	0.49
2205175119	0.93	0.26	0.221	0.59	0.67
2205175121	0.77	0.41	0.102	0.59	0.36
2205175124	0.86	0.43	0.296	0.64	0.43
2205175125	0.82	0.28	0.069	0.55	0.54
2205175130	0.89	0.21	0.094	0.55	0.68
2205175133	0.93	0.53	0.083	0.73	0.40
2205175134	0.72	0.07	0.048	0.40	0.65
2205175141	1.18	0.81	0.382	0.99	0.37
2205175147	0.87	0.27	0.194	0.57	0.60
2205175192	1.53	1.01	0.467	1.27	0.52
2205175193	0.67	0.38	0.258	0.53	0.29
2205183122	0.87	0.61	0.155	0.74	0.26
2205183123	1.02	0.33	0.024	0.67	0.69
2205183125	0.82	0.43	0.204	0.63	0.39
2205183126	0.94	0.47	0.308	0.71	0.47
2205183128	0.65	0.23	0.159	0.44	0.42
2205183129	1.49	1.01	0.092	1.25	0.48
2205183130	1.65	0.93	0.065	1.29	0.72
2205183131	0.98	0.35	0.083	0.66	0.63
2205183133	0.95	0.63	0.151	0.79	0.32
2205183134	0.7	0.26	0.045	0.48	0.44
2205183135	0.84	0.41	0.441	0.63	0.43
2205183137	0.72	0.71	0.711	0.72	0.01
2205183138	0.79	0.28	0.011	0.54	0.51
2205183139	0.65	0.40	0.440	0.53	0.25
2205183141	1.07	0.95	0.249	1.01	0.12
2205183142	1.25	0.27	0.194	0.76	0.98
2205183143	0.79	0.29	0.016	0.54	0.50
2205183144	1.4	1.42	0.252	1.41	-0.02

2205183146	1.06	0.18	0.063	0.62	0.88
2205183147	1.08	0.57	0.393	0.83	0.51
2205183149	1.69	0.05	0.024	0.87	1.64
2205183151	1.11	0.57	0.750	0.84	0.54
2205183152	0.69	0.21	0.227	0.45	0.48
2205183153	0.64	0.30	0.287	0.47	0.34
2205183154	0.83	0.18	0.115	0.51	0.65
2205183155	0.89	0.12	0.040	0.51	0.77
2205183157	0.85	0.33	0.197	0.59	0.52
2205183158	0.96	0.39	0.192	0.68	0.57
2205183159	0.82	0.08	0.013	0.45	0.74
2205183160	0.8	0.62	0.851	0.71	0.18
2205183161	0.9	0.44	0.468	0.67	0.46
2205183162	0.86	0.88	0.061	0.87	-0.02
2205183163	0.77	1.10	1.201	0.93	-0.33
2205183164	0.77	0.16	0.123	0.46	0.61
2205183165	0.76	0.32	0.064	0.54	0.44
2205183166	0.71	1.25	1.041	0.98	-0.54
2205183167	0.93	0.15	0.098	0.54	0.78
2205183168	0.96	0.03	0.007	0.49	0.93
2205183169	0.82	0.53	0.072	0.67	0.29
2205183170	0.7	0.55	0.063	0.63	0.15
2205183172	0.67	0.60	0.339	0.63	0.07
2205183173	0.8	0.32	0.109	0.56	0.48
2205183174	0.73	0.95	0.460	0.84	-0.22
2205183175	0.99	0.29	0.064	0.64	0.70
2205183176	1.22	0.60	0.448	0.91	0.62
2205183177	0.83	0.31	0.064	0.57	0.52
2205183178	1.08	0.64	0.150	0.86	0.44
2205183180	0.64	0.43	0.369	0.53	0.21
2205183181	0.78	0.39	0.125	0.58	0.39
2205183182	0.64	0.22	0.226	0.43	0.42
2205183184	0.83	0.20	0.000	0.52	0.63
2205183185	0.84	0.78	0.452	0.81	0.06
2205183186	0.84	0.20	0.094	0.52	0.64
2205183187	0.97	0.62	0.308	0.80	0.35
2205183188	0.84	0.25	0.178	0.55	0.59
2205183189	0.78	0.28	0.115	0.53	0.50
2205183190	0.91	0.26	0.103	0.58	0.65
2205183192	1.08	0.56	0.162	0.82	0.52
2205183193	0.96	0.88	0.022	0.92	0.08
2205183194	0.88	0.75	0.112	0.82	0.13
2205183195	0.64	0.57	0.128	0.61	0.07
2205183196	0.83	0.59	0.691	0.71	0.24
2205183198	0.64	0.38	0.284	0.51	0.26

2205183199	0.99	0.71	0.773	0.85	0.28
2205183201	0.86	0.29	0.176	0.58	0.57
2205183202	0.69	0.36	0.006	0.53	0.33
2205183203	0.78	0.82	0.118	0.80	-0.04
2205183204	1.08	1.02	0.196	1.05	0.06
2205183206	0.62	0.51	0.168	0.56	0.11
2205183207	0.73	0.64	0.239	0.68	0.09
2205183208	0.79	0.72	0.874	0.75	0.07
2205183209	0.88	0.59	0.417	0.73	0.29
2205183210	0.99	0.97	0.197	0.98	0.02
2205183211	0.99	0.60	0.060	0.79	0.39
2205183212	0.91	0.31	0.139	0.61	0.60
2205183213	0.99	0.21	0.249	0.60	0.78
2205183214	1	1.11	0.086	1.05	-0.11
2205183216	0.78	0.34	0.132	0.56	0.44
2205183217	0.95	0.42	0.195	0.69	0.53
2205183218	0.59	0.29	0.013	0.44	0.30
2205305132	0.93	0.21	0.131	0.57	0.72
2205305133	1	0.48	0.170	0.74	0.52
2205305134	0.86	0.43	0.025	0.65	0.43
2205305135	0.98	0.54	0.163	0.76	0.44
2205305136	0.88	0.41	0.462	0.65	0.47
2205305137	1.17	0.43	0.279	0.80	0.74
2205305138	0.79	0.32	0.077	0.56	0.47
2205305139	1.02	0.14	0.037	0.58	0.88
2205305140	0.77	0.27	0.050	0.52	0.50
2205305141	1	0.97	0.660	0.98	0.03
2205305142	1.12	0.68	0.294	0.90	0.44
2205305143	0.98	0.49	0.003	0.74	0.49
2205305144	0.67	0.51	0.654	0.59	0.16
2205305145	1.17	0.26	0.177	0.72	0.91
2205305146	0.86	0.25	0.205	0.55	0.61
2205305147	0.74	0.28	0.051	0.51	0.46
2205305148	1.1	0.50	0.070	0.80	0.60
2205305149	0.82	0.13	0.090	0.47	0.69
2205305150	0.76	0.42	0.107	0.59	0.34
2205305151	0.92	0.23	0.187	0.58	0.69
2205305152	0.78	0.28	0.032	0.53	0.50
2205305153	1.11	0.32	0.073	0.72	0.79
2205305154	1.11	0.38	0.524	0.75	0.73
2205305155	1.03	0.38	0.127	0.71	0.65
2205305156	0.97	0.45	0.074	0.71	0.52
2205305157	1.33	0.65	0.007	0.99	0.68
2205305158	1.42	0.88	0.142	1.15	0.54
2205305159	0.78	1.02	0.945	0.90	-0.24

2205305160	0.78	0.40	0.521	0.59	0.38
2205305161	0.92	0.44	0.027	0.68	0.48
2205305162	0.96	0.14	0.152	0.55	0.82
2205305163	1.36	0.28	0.049	0.82	1.08
2205305164	0.89	0.38	0.486	0.63	0.51
2205305165	0.96	0.95	0.264	0.96	0.01
2205305166	0.96	0.11	0.098	0.53	0.85
2205305167	1.76	1.66	0.433	1.71	0.10
2205305168	0.97	0.29	0.277	0.63	0.68
2205305169	1.04	0.61	0.335	0.82	0.43
2205305170	1.22	0.91	0.311	1.06	0.31
2205305171	1.24	0.81	0.700	1.03	0.43
2205305172	0.74	0.23	0.309	0.48	0.51
2205305173	0.97	0.43	0.452	0.70	0.54
2205305174	0.96	0.21	0.027	0.59	0.75
2205305175	0.69	0.15	0.181	0.42	0.54
2205305176	0.89	0.20	0.185	0.54	0.69
2205305177	1.06	0.60	0.637	0.83	0.46
2205305178	0.78	0.39	0.040	0.58	0.39
2205305179	0.99	0.25	0.322	0.62	0.74
2205305180	0.91	0.05	0.060	0.48	0.86
2205305181	0.86	0.65	0.496	0.76	0.21
2205305182	1.38	0.75	0.604	1.07	0.63
2205305183	0.97	0.07	0.048	0.52	0.90
2205305184	0.74	0.15	0.152	0.45	0.59
2205305185	0.93	0.25	0.332	0.59	0.68
2205305186	0.49	0.18	#DIV/0!	0.33	0.31
2205305187	0.78	0.80	0.915	0.79	-0.02
2205305188	1.28	0.65	0.312	0.97	0.63
2205305189	1.37	0.53	0.223	0.95	0.84
2205305190	0.67	0.04	0.055	0.36	0.63
2205305191	1.19	1.24	0.547	1.21	-0.05
2205105037	1.33	0.55	0.088	0.94	0.78
2205115041	1.3	0.65	0.415	0.98	0.65
2205115026	5.86	5.83	0.631	5.84	0.03
2205115035	3.69	2.85	1.272	3.27	0.84
2205115037	3.98	2.63	0.115	3.30	1.35
2205115042	4.09	3.42	0.456	3.75	0.67
2205115059	6.19	3.87	0.610	5.03	2.32
2205115060	4.94	4.17	0.918	4.56	0.77
2205115063	4.35	4.42	1.654	4.39	-0.07
2205115069	4.59	3.94	0.369	4.26	0.65
2205115071	4.59	3.00	0.489	3.80	1.59
2205169751	5.46	4.19	0.686	4.83	1.27
2205169755	4.39	4.20	0.801	4.29	0.19

2205169757	4.31	2.71	0.008	3.51	1.60
2205169761	6.16	5.22	1.083	5.69	0.94
2205169764	5.75	4.65	1.867	5.20	1.10
2205169770	5.12	4.92	2.606	5.02	0.20
2205169778	5.26	3.79	0.871	4.53	1.47
2205169783	4.29	3.95	0.988	4.12	0.34
2205175176	5.52	3.85	0.456	4.68	1.67
2205183215	4.08	3.78	2.134	3.93	0.30
2205309290	3.41	0.99	0.887	2.20	2.42
2205309292	4.55	2.49	0.602	3.52	2.06
2205309294	3.73	2.73	0.150	3.23	1.00
2205309296	5.2	5.05	1.579	5.12	0.15
2205309297	4.01	4.47	0.247	4.24	-0.46
2205309300	4.13	3.76	1.832	3.95	0.37
2205309302	4.28	4.24	0.513	4.26	0.04
2205309305	4.81	4.44	0.540	4.63	0.37
2205309307	5.17	4.02	0.385	4.59	1.15
2205309308	5.01	4.68	0.347	4.84	0.33
2205309309	4.78	3.68	0.651	4.23	1.10
2205315403	4.32	4.15	0.021	4.24	0.17
average					0.51
SD					0.39
